# The roles of long noncoding RNA-mediated macrophage polarization in respiratory diseases

**DOI:** 10.3389/fimmu.2022.1110774

**Published:** 2023-01-05

**Authors:** Xin Qiao, Yuxiao Ding, Dasen Wu, Anle Zhang, Yan Yin, Qiuyue Wang, Wei Wang, Jian Kang

**Affiliations:** Department of Pulmonary and Critical Care Medicine, The First Hospital of China Medical University, Shenyang, China

**Keywords:** macrophages, M1/M2 polarization, long noncoding RNAs, respiratory diseases, lung cancer

## Abstract

Macrophages play an essential role in maintaining the normal function of the innate and adaptive immune responses during host defence. Macrophages acquire diverse functional phenotypes in response to various microenvironmental stimuli, and are mainly classified into classically activated macrophages (M1) and alternatively activated macrophages (M2). Macrophage polarization participates in the inflammatory, fibrotic, and oncogenic processes of diverse respiratory diseases by changing phenotype and function. In recent decades, with the advent of broad-range profiling methods such as microarrays and next-generation sequencing, the discovery of RNA transcripts that do not encode proteins termed “noncoding RNAs (ncRNAs)” has become more easily accessible. As one major member of the regulatory ncRNA family, long noncoding RNAs (lncRNAs, transcripts >200 nucleotides) participate in multiple pathophysiological processes, including cell proliferation, differentiation, and apoptosis, and vary with different stimulants and cell types. Emerging evidence suggests that lncRNAs account for the regulation of macrophage polarization and subsequent effects on respiratory diseases. In this review, we summarize the current published literature from the PubMed database concerning lncRNAs relevant to macrophage polarization and the underlying molecular mechanisms during the occurrence and development of respiratory diseases. These differentially expressed lncRNAs are expected to be biomarkers and targets for the therapeutic regulation of macrophage polarization during disease development.

## 1 Introduction

Respiratory diseases are responsible for a significant proportion of serious morbidity and premature death worldwide (1). The Global Burden of Diseases (GBD) Study 2017 data showed that there were 3.2 million deaths due to COPD and 495,000 deaths due to asthma (2). Lung cancer is the deadliest of nearly all cancers, with 5-year survival rates of 4-17% depending on stage and regional differences (3). In addition, the COVID-19 pandemic has claimed more than 5.7 million lives within a year, mostly from respiratory causes (4). A number of other conditions, including interstitial disease diagnosed as IPF, noninfectious granulomatous lung diseases such as sarcoidosis, and infectious lung diseases such as tuberculosis, also contribute to a significant global burden. Therefore, early diagnosis and precise therapy are especially crucial. Despite considerable advancements in fundamental research and clinical practice that have shed light on the pathophysiology of these diseases in recent decades, challenges remain in exploring molecular mechanisms.

Macrophages play an essential role in maintaining the normal function of the innate and adaptive immune responses during host defence (5). Macrophages acquire diverse functional phenotypes in response to various microenvironmental stimuli, which are mainly classified into classically activated macrophages (M1) and alternatively activated macrophages (M2) (6). Exposure to interferon-γ (IFN-γ) or lipopolysaccharide (LPS) stimulates M1 macrophage polarization with the ability to produce proinflammatory cytokines such as IL-1β, IL-6, IL-12, and TNFα, leading to pathogen clearance and tissue damage (7). In contrast, M2-polarized macrophages are further classified into four subsets based on their *in vitro* responses to stimuli: M2a macrophages (characterized by the expression of CD206 receptors on the cell surface and that can be induced by IL-4 and IL-13), which promote type II immune responses and fibrogenesis; M2b macrophages (characterized by the expression of CD86 receptors on the cell surface and that can be induced by immune complexes), which are immunoregulatory; M2c macrophages (characterized by the expression of CD163 receptors on the cell surface and that can be induced by IL-10 and transforming growth factor-β (TGF-β), which are anti-inflammatory and initiators of tissue remodelling; and M2d macrophages, also known as tumour-associated macrophages (TAMs), which are the major inflammatory component of the tumour microenvironment (TME) (7–11). The pathogenic process of most respiratory diseases has been proposed to be regulated by macrophage plasticity (M1/M2 polarization) (12). For example, the activation of M1 macrophage polarization is important for inflammation formation, while the activation of M2 macrophage polarization is important for fibrosis, inflammation solution and tumorigenesis (12, 13). In this scenario, targeting the balance of macrophage phenotypes may be a key step in respiratory disease management.

In recent decades, with the advent of broad-range profiling methods such as microarrays and next-generation sequencing, the discovery of RNA transcripts that do not encode proteins termed “noncoding RNAs (ncRNAs)” has become more easily accessible. NcRNAs are generally classified into two groups, housekeeping and regulatory ncRNAs, according to their regulatory effects. As one major member of the regulatory ncRNA family, long noncoding RNAs (transcripts >200 nucleotides) participate in multiple pathophysiological processes, including cell proliferation, differentiation and apoptosis, which vary with different stimulants and cell types (14, 15). Many studies have reported that the lncRNA profiles in patients with respiratory disease differ from those in healthy people (16–18). In addition, emerging evidence suggests that dysregulated lncRNAs account for the pathogenesis and progression of several lung diseases, including COPD, asthma, and ALI, due to their roles in regulating macrophage polarization (19–21). In this review, we will summarize recent findings regarding lncRNA-mediated macrophage polarization in four categories of respiratory conditions: chronic airway disease (asthma, COPD, and cystic fibrosis), interstitial lung disease (IPF, CTD-ILD, and sarcoidosis), infectious lung disease (TB, pneumonia, and acute lung injury/acute respiratory distress syndrome), and lung cancer, providing a theoretical basis for the use of lncRNAs as noninvasive diagnostic biomarkers and therapeutic targets for respiratory diseases.

## 2 LncRNA expression profiles in M1/M2 macrophage polarization

A number of studies have analysed the expression profiles of lncRNAs in human- and murine-derived macrophages under various polarized conditions. Zhang et al. (22) used murine bone marrow-derived macrophages (BMDMs) to determine lncRNA expression in M1 and M2 polarizing conditions. In this study, M1-polarised conditions in BMDMs were induced by LPS plus IFN-γ treatment, whereas M2 polarization was stimulated by IL-4. The lncRNA-microarray results identified 33,231 lncRNAs, of which 627 lncRNAs were enriched in M1 macrophages and 624 lncRNAs were enriched in M2 macrophages with the selection criteria of >2-fold differentially expressed changes and FDR adjusted P values < 0.05 (22). Using qRT−PCR, they confirmed that in M1 polarized macrophages, *lncAK048798* and *lncAK153212* were downregulated, whereas *lncAK085865* and *lncAK083884* were upregulated in comparison to levels in M2 polarized conditions (22). Luo and his colleague (23) used microarray analyses to analyse the expression of lncRNAs in the process of M2 to M1 macrophage polarization in human monocytic U937 cells. Specifically, stimulation of U937 cell cultures with PMA, IL-4, and IL-13 induced the M2 phenotype, while a switch from the M2 to the M1 phenotype was promoted by LPS and IFN-γ stimulation. They uncovered 26,276 differentially expressed lncRNAs between M1 and M2 phenotypes of U937 macrophages. Pearson correlation analysis was used to verify the agreement between microarray data and qRT−PCR examination. Although the qRT−PCR results of some lncRNAs were inconsistent with the microarray results, the majority of lncRNAs analysed were congruent in both assays. On the other hand, Ito et al. (24) stimulated mouse BMDMs into various phenotypes with IFN-γ (M1), IL-4 (M2a), LPS and immobilized IgG (M2b), and IL-10 (M2c). The qRT−PCR results showed that lncRNA growth arrest specific 5 (*GAS5*) is not expressed by M2b cells, but M0, M1, M2a, and M2c cells express it. Additionally, BMDMs overexpressing *GAS5* RNA after *GAS5* gene transduction did not switch to the M2b phenotype after stimulation with LPS and IC in combination.

**Figure 1:**
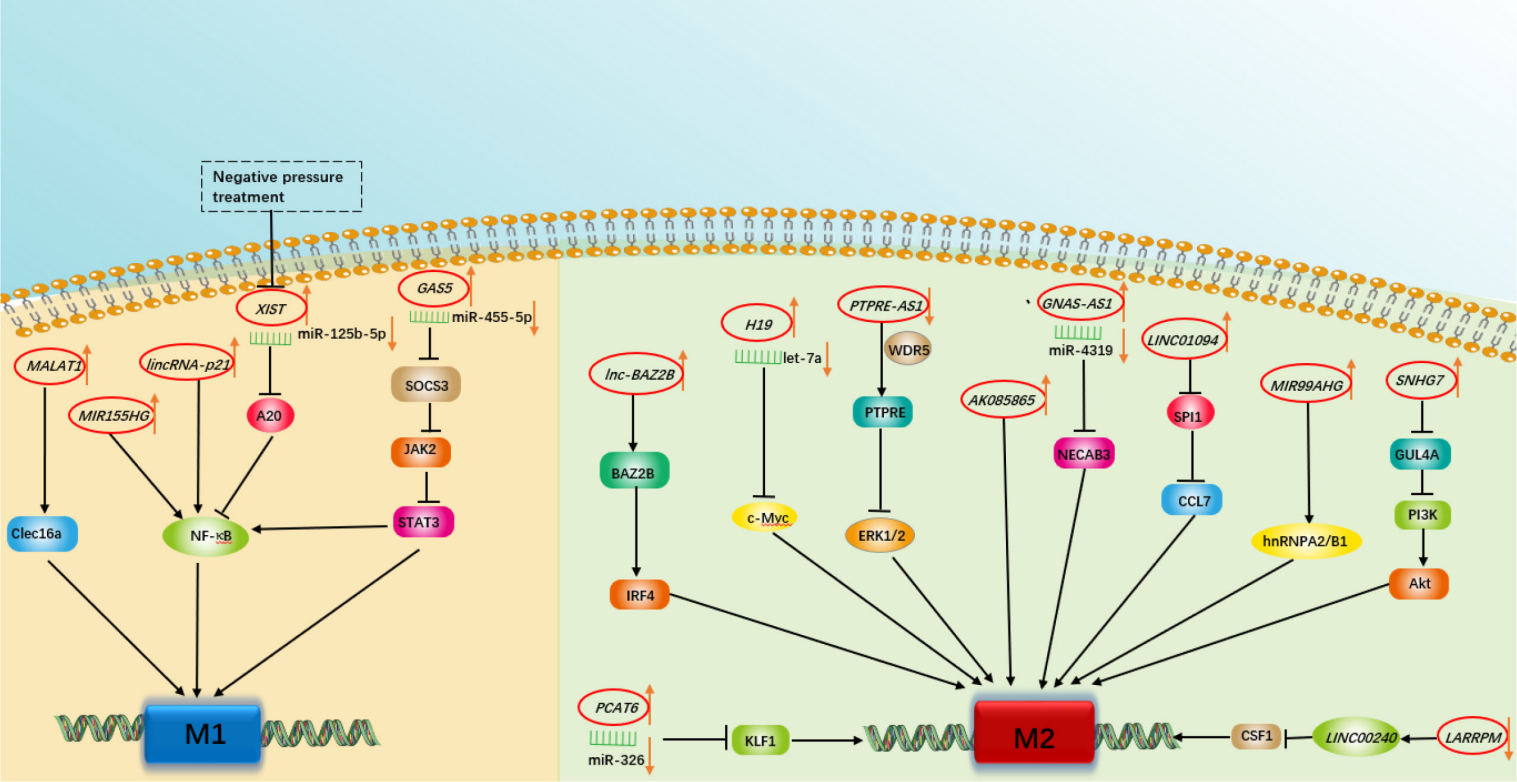
A schematic summary of the role of various long non-coding RNAs in modulating macrophage polarization involved in respiratory diseases.

In summary, profiling lncRNA expression in polarized macrophages with techniques such as microarray and RT−qPCR arrays yields large amounts of dysregulated lncRNAs. For the most part, the therapeutic potential of such dysregulated lncRNAs through regulating macrophage polarization is worth exploring. In the following sections, we summarize the broad spectrum of lncRNAs involved in macrophage polarization along with their target proteins and their possible roles in the regulation of respiratory diseases. An overview of these lncRNAs is given in **Table 1** and **Figure 1**.

**Table 1:** LncRNAs regulate M1/M2 polarization through targeting various adaptor proteins and transcription factors in respiratory diseases.

Type of diseases	LncRNAs	Cell types	Polarization	Targets	Function	Reference
Asthma	*AK085865*	Primary macrophages in the BALF from mouse	Promote M2	None	*AK085865* knockout ameliorates asthmatic airway inflammation.	Pei et al. (20)
	*PTPRE-AS1*	Bone marrow-derived macrophages (BMDMs) and RAW 264.7	Suppress M2	PTPRE/ERK1/2	Protects against allergic inflammation.	Han et al. (25)
	*Lnc-BAZ2B*	PMA-induced human monocyte THP-1 cells	Promote M2	BAZ2B/IRF4	Enhances the disease severity of allergic asthma.	Xia et al. (17)
Chronic obstructive pulmonary disease (COPD)	*MIR155HG*	GM-CSF induced peripheral blood mononuclear cells (PBMCs)	Promote M1, suppress M2	NF- ĸB/p65	Enhance pro-inflammatory cytokine release.	Li et al. (19)
Idiopathic Pulmonary Fibrosis (IPF)	*H19*	THP-1 macrophages and BMDMs	Promote M2	let-7a/c-Myc	Promotes myofibroblast differentiation.	Xiao et al. (26)
Pulmonary tuberculosis	*XIST*	RAW264.7 cells and human monocyte-derived macrophages (hMDMs)	Suppress M1	miR-125b-5p/A20/NF-κB	*XIST* downregulation suppress preexisting MTB infection.	Luo et al. (27)
	*MIR99AHG*	PBMCs, monocyte-derived macrophages (MDMs), BMDMs	Promote M2	hnRNPA2/B1	Promote MTB growth.	Gcanga et al. (28)
Pneumonia	*GAS5*	HMDMs and PBMCs	Promote M1	miR-455-5p/SOCS3/JAK2/STAT3	Protect against childhood pneumonia.	Chi et al. (29)
Acute lung injury (ALI)/ Acute respiratory distress syndrome (ARDS)	*LincRNA-p21*	MH-S	Promote M1	NF-κB/p65	*LincRNA-p21* inhibition may protect against ALI.	Zhang et al. (21)
	*MALAT1*	Mouse BMDMs, human PBMCs and THP-1 macrophages and mouse alveolar macrophages	Promote M1	Clec16a	*Malat1* knockout ameliorated LPS-induced pulmonary inflammation and injury but led to severe lung fibrosis.	Cui et al. (30)
Lung cancer	*GNAS-AS1*	PMA-induced human monocyte THP-1 cells	Promote M2	miR-4319/NECAB3	Facilitating the progression of NSCLC.	Li et al. (31)
	*LARRPM*	PMA-induced human monocyte THP-1 cells	Suppress M2 Promote M1	*LINC00240*/CSF1	Suppressed LUAD cell proliferation, migration and invasion, and promoted apoptosis.	Li et al. (32)
	*LINC01094*	PMA-induced human monocyte THP-1 cells	Promote M2	SPI1/CCL7	Facilitating the progression of LUAD.	Wu et al. (33)
	*PCAT6*	PMA-induced human monocyte THP-1 cells	Promote M2	miR-326/KLF1	Promoted metastasis and EMT process of NSCLC cells.	Chen et al. (34)
	*SNHG7*	THP-1 macrophages	Promote M2	CUL4A/PTEN/PI3K/Akt	Enhanced docetaxel resistance of LUAD cells.	Zhang et al. (35)

## 3 LncRNA-based regulation of macrophage polarization in chronic airway disease

Chronic airway diseases, characterized by airway inflammation and airway remodelling, are increasing as a cause of morbidity and mortality for all age groups and races across the world. The underlying molecular mechanisms involved in chronic inflammatory airway diseases have not been fully explored. Recently, accumulative evidence has shown that the novel regulatory mechanism underlying the action between lncRNAs and polarized macrophages plays a critical role in the pathophysiological processes of chronic airway diseases, particularly chronic obstructive pulmonary disease (COPD) and asthma.

### 3.1 Asthma

**Figure 2:**
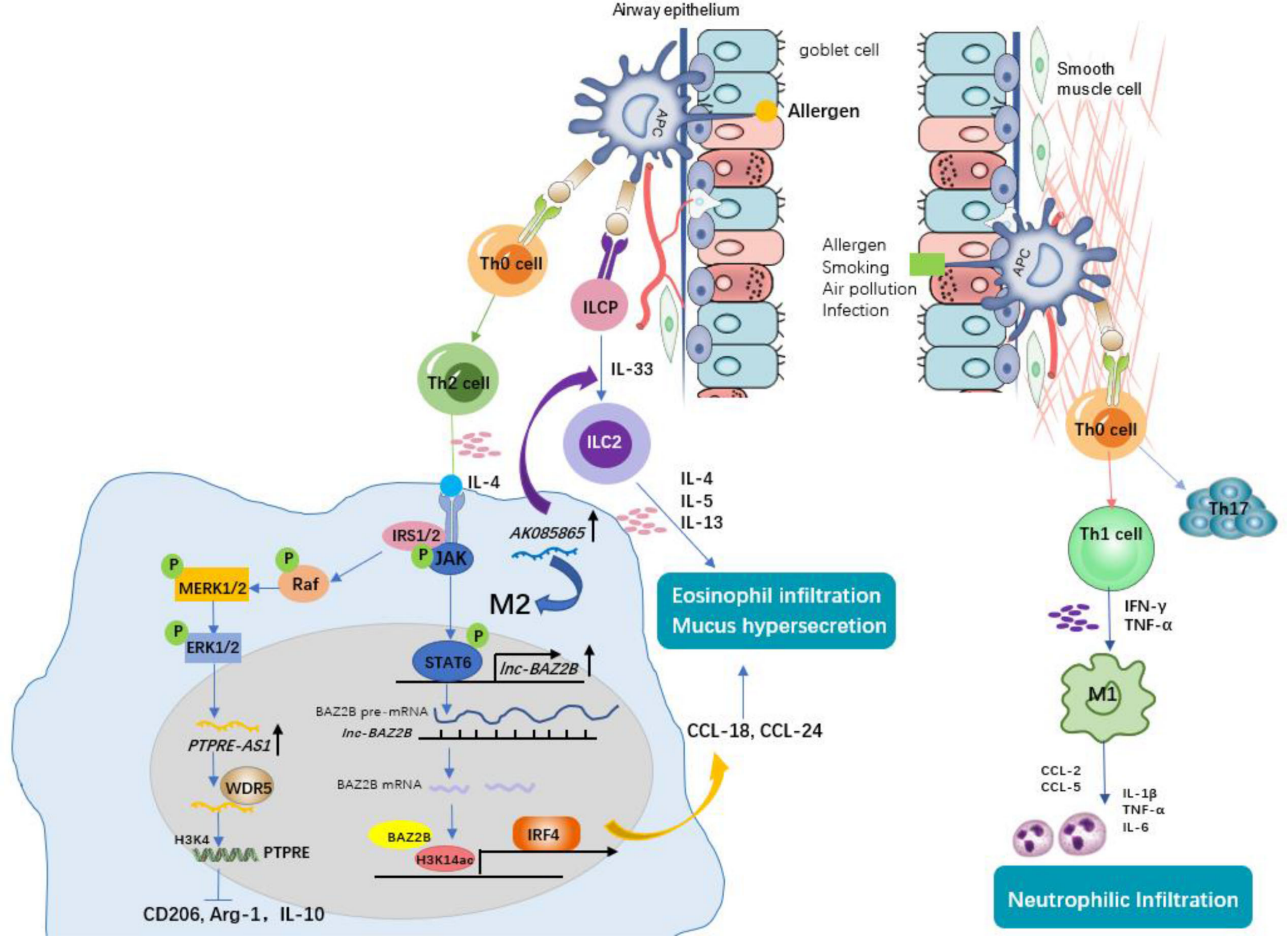
The role of macrophages and associated lncRNAs in allergic asthma. Antigen-presenting cells (APCs) identify allergens and initiate the allergic response. Under this condition, naïve CD4+ T cells differentiate into Th2 cells. Th2 cells secreting IL-4 induce M2 macrophage polarization. *Lnc-BAZ2B* promotes M2 macrophage activation by stabilizing BAZ2B pre-mRNA, thereby promoting IRF4 expression and chemokines secretion, leading to mucus hypersecretion and eosinophil infiltration in allergic children. *PTPRE-AS1* is selectively expressed in IL-4–stimulated M2 macrophages. *PTPRE-AS1* binds WDR5 directly, modulating H3K4me3 of the PTPRE promoter to regulate PTPRE-dependent signaling during M2 macrophage activation and protects against pulmonary allergic inflammation. LncRNA *AK085865* promotes M2 macrophages activation and M2 macrophages promote the differentiation of innate lymphoid cells progenitor (ILCP) into type II innate lymphoid cells (ILC2s), thus aggravate Type 2 immune response. Non-Th2 asthma (i.e., neutrophilic asthma), by contrast, is driven by M1 macrophages or Th1/Th17 lymphocytes through the production of high levels of proinflammatory Th1 cytokines (e.g., IL-6, IL-1β, and TNF-α) and chemokines (e.g., CCL2 and CCL5), contributing to neutrophilic infiltration and airway inflammation.

Asthma, characterized by reversible airflow limitation, airway inflammation and airway hyperresponsiveness, is mainly divided into two phenotypes: Th2 and non-Th2 (36, 37). Th2-asthma (i.e., eosinophilic asthma) is well established to play a leading role in asthma development, as more than half of asthma cases have a Th2 phenotype, where M2 macrophages predominantly secrete high levels of IL-13 and chemokines (e.g., CCL-17 and CCL-18), inducing airway eosinophil infiltration and mucus hypersecretion and contributing to lung function impairment and airway remodelling (38, 39). Non-Th2 asthma (i.e., neutrophilic asthma), by contrast, is characterized by neutrophil dominance airway inflammation that can be driven by M1 macrophages or Th1/Th17 lymphocytes and by the production of high levels of proinflammatory Th1 cytokines (e.g., IL-6, IL-1β, and TNF-α) and chemokines (e.g., CCL2 and CCL5) (36, 38, 40). Thus, patients with neutrophilic asthma have a poor response to corticosteroids and tend to develop severe or refractory asthma (41, 42). These results indicate that regulating the M1 and M2 macrophage phenotype balance would guide individualized therapy for different types of asthma. To date, several lncRNAs have been proven to play an important role in the pathogenesis of asthma by regulating M1/M2 macrophage polarization balance (**Figure 2**). We discuss them below.

Pei and his colleagues (20) found that there was high expression of lncRNA *AK085865* in BAL cells and lung tissues from dermatophagoides farinae protein 1 (Der f1)-induced asthmatic mice compared with PBS-induced mice. LncRNA *AK085865* expression was upregulated during the M1 to M2 transition (20). Upon *AK085865* knockdown, IgE-mediated eosinophilic airway inflammation and M2 macrophages were both decreased in asthmatic mice, which proved that *AK085865* knockout protected against allergic inflammation in mice by inhibiting M2 polarization. Furthermore, *AK085865* could promote the differentiation of innate lymphoid cells progenitor (ILCP) into type II innate immune lymphoid cells (ILC2s) and then augment type 2 inflammation (20). Therefore, knockout of the lncRNA *AK085865* may guide novel treatment for type 2 asthma. Xia et al. (17) conducted a differential lncRNA expression profile and found that lnc-*BAZ2B* was upregulated and that its expression was correlated with BAZ2B expression in the PBMCs of children with asthma. In addition, *lnc-BAZ2B* knockdown significantly inhibited the M2-specific marker expression of THP1-derived macrophages *in vitro*. Further mechanistic investigation showed that *lnc-BAZ2B* was an upstream regulator of BAZ2B and positively regulated the expression of BAZ2B by stabilizing its pre-mRNA, which promoted the transcription of IRF4 by binding H3K14ac-modified sites within the IRF4 gene and thus influenced the activation of M2 macrophages (17). This observation was consistent with a cockroach allergen extract (CRE)-induced asthma model, where BAZ2B knockdown inhibited pulmonary inflammation and mucus secretion by inhibiting M2 macrophage polarization *via* IRF4 (17). Taken together, we speculate that inactivation of *lnc-BAZ2B* could help prevent Th2 asthma aggravation. Another lncRNA reported to be upregulated during IL-4–induced M2 macrophage activation is *PTPRE-AS1* (25). Knockdown of *PTPRE-AS1* expression promoted transcription of M2 marker genes by targeting receptor-type tyrosine protein phosphatase (PTPRE), which promoted IL-4–induced activation of MAPK/ERK 1/2 signalling (25). Moreover, *PTPRE-AS1* plays a positive role in the regulation of PTPRE expression and protects against allergic inflammation by inhibiting M2 macrophage polarization, whether in a mouse model or in PBMCs from asthmatic patients, whereas it promotes M1-associated colitis functionally (25). Additionally, *PTPRE-AS1* can be used to distinguish asthma patients from normal individuals by receiver operating curve (ROC) analysis, implying that *PTPRE-AS1* exhibits potential as a biomarker in childhood asthma (25). During M2 macrophage activation, *PTPRE-AS1* directly bound to WDR5, regulating PTPRE-dependent signalling by modulating the PTPRE promoter’s H3K4me3. These results provide evidence to support the potential of lncRNA *PTPRE-AS1* to serve as a biomarker for type 2 inflammation remission.

Taken together, these data show that lncRNAs possess wonderful capability as biomarkers and therapeutic targets for asthma. This potential, however, remains far from being completely assessed. For example, most published lncRNAs are focused on regulating M2 macrophage-mediated type 2 inflammation, and identifying those lncRNA profiles that can regulate M1 macrophages is essential for confirming the potential of lncRNAs to identify asthma phenotype and determine the optimal treatment for each patient. Therefore, there are many opportunities for further research.

### 3.2 COPD

**Figure 3:**
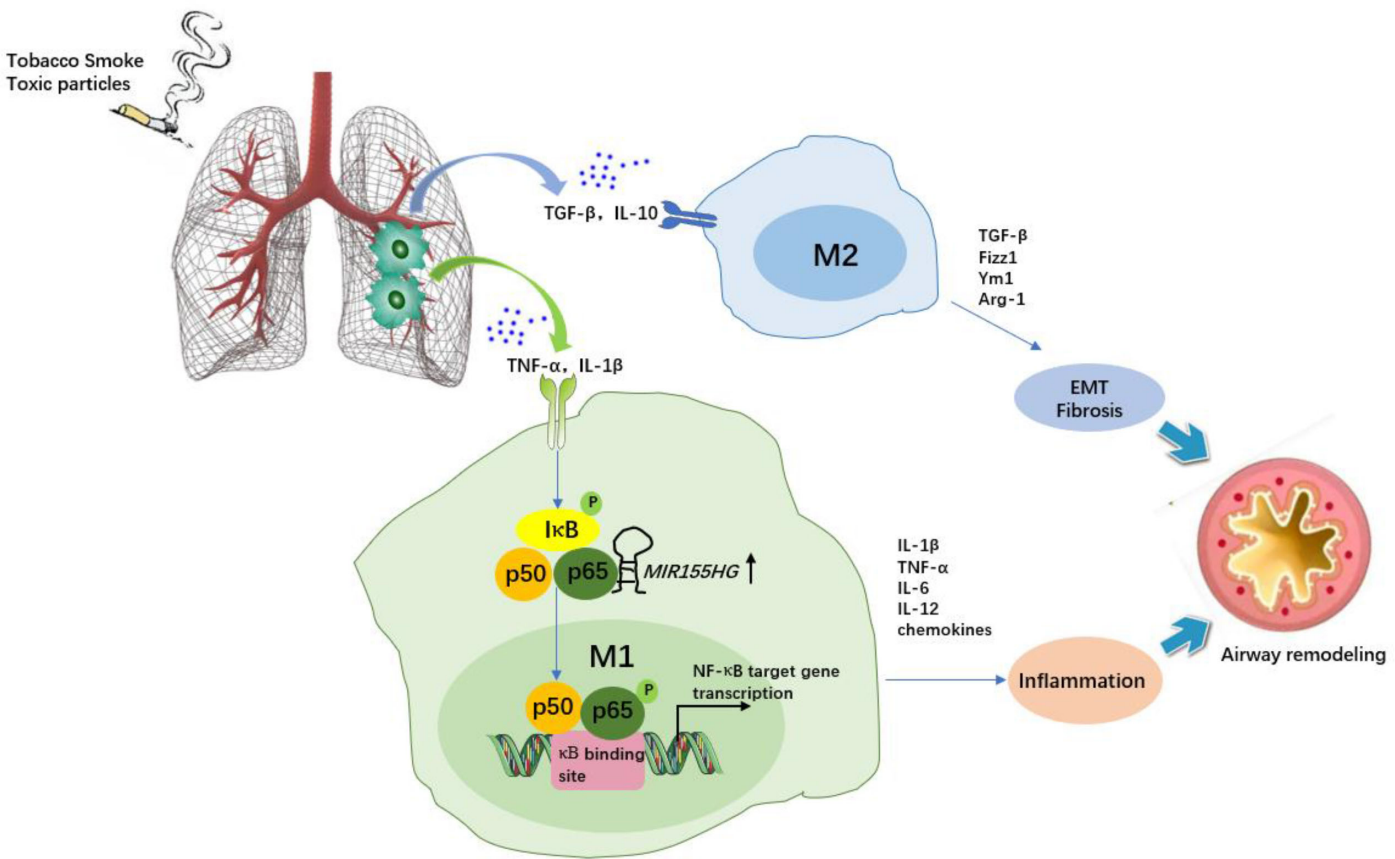
The role of macrophages and *MIR155HG* in chronic obstructive pulmonary disease (COPD). Tobacco smoke or toxic particles cause airway inflammation and airway remodelling. Macrophage polarization plays a key role. M1 macrophages secrete inflammatory cytokines and chemokines, promoting neutrophil recruitment and augmenting airway inflammation. *MIR155HG* indirectly regulate NF-κB signaling activity by interacting with the p65-p50 complex, resulting in M1 macrophages activation accompanied by enhanced proinflammatory cytokine release (TNF-α, IL-1β and IL-12). M2 macrophage-induced TGF-β, Fizz1 and Ym1 are involved in epithelial mesenchymal transformation (EMT) and extracellular matrix dynamics, and arginine accelerates collagen synthesis, which leads to fibrosis.

COPD is a preventable and treatable condition characterized by persistent airflow restriction and chronic airway inflammation (43). Cigarette smoking (CS) is the largest known risk factor for COPD (43). The phenotypic transformation of macrophages induced by CS or cigarette smoking extract (CSE) has been demonstrated in *in vivo* and *in vitro* studies of COPD (44–47). In addition to CS exposure, biomass ambient particulate matter (PM) has been indicated to be crucial for COPD pathogenesis by numerous epidemiological studies. Recent studies have shown that biomass fuel smoke (BMF) and PM2.5 facilitate macrophage polarization and activation *in vitro* (48, 49). Consistently, M1 and M2 macrophages have been detected in the lungs of COPD patients (46). Functionally, M1 macrophage-induced iNOS, IL-1β, IL-6, IL-8, and TNF-α contribute to oxidative stress and airway inflammation, while M2 macrophage-induced TGF-β facilitates epithelial–mesenchymal transition (EMT)-based small airway remodelling in COPD (46, 47, 50). In addition, M2 macrophage-induced TGF-β, Fizz1 and Ym1 are both involved in extracellular matrix dynamics, and arginine accelerates collagen synthesis, which leads to fibrosis (51). Therefore, the functional differentiation of macrophages is important for the specific pathology observed in COPD (**Figure 3**).

The MIR155 host gene (*MIR155HG*), an endogenous lncRNA located at chromosome 21q2 within a 13-kb length, is upregulated in M1 macrophages (52, 53), but *MIR155HG* promoter activity could be inhibited by M2 macrophage-secreted IL-10 in a STAT3-dependent manner (signal transducer and activator of transcription 3) *via* its Ets1 transcription factor-binding site (54). Li and his colleagues (19) recently found that *MIR155HG* was highly expressed in peripheral blood mononuclear cells of COPD patients compared with normal controls, and *MIR155HG* overexpression resulted in a significantly increased percentage of M1 macrophages accompanied by enhanced proinflammatory cytokine release (TNF-α, IL-1β and IL-12) as well as decreased M2 macrophage levels, whereas interference with *MIR155HG* expression reduced the ratio of M1/M2 macrophages (19). Mechanistically, the NF-κB protein p65 could combine with the upstream transcription site of *MIR155HG*, suggesting that *MIR155HG* was a direct NF-κB target gene contributing to airway inflammation in COPD (**Figure 3**). Another report also showed increased expression of *MIR155HG* in lung tissues of smokers with non-COPD or COPD compared to healthy controls, particularly in COPD patients (55). Therefore, targeting *MIR155HG* to reduce M1 differentiation may be beneficial for COPD relief. On the other hand, *MIR155HG* is also known for promoting cell migration, proliferation, and invasion in multiple cancers, including non-small cell lung cancer (NSCLC), where M2d macrophages (TAMs) play a key role in tumorigenesis (56, 57). This might contradict the regulatory role of *MIR155HG* in COPD. The exact effect of targeting *MIR155HG* to shift the macrophage phenotype in COPD complicated with lung cancer should be considered. Indeed, macrophage polarization is a complex and dynamic evolutionary process that is affected by various environmental stimuli. These findings offer multiple chances to further explore the role of *MIR155HG* in respiratory diseases.

### 3.3 Cystic fibrosis

CF is a major disease associated with bronchiectasis and is caused by a mutation in a gene called cystic fibrosis transmembrane conduction regulator (CFTR), whose dysfunction gives rise to mucus retention, chronic infection, and airway inflammation (58). Human monocytes, alveolar macrophages, and monocyte-derived macrophages (MDMs) express CFTR protein, and CFTR dysfunction in macrophages is related to augmented airway inflammation, increased mucus cells and mucus blockage, and increased lethal pneumonia, emphasizing the critical role of macrophages in promoting CF (59–61). Most importantly, Tarique et al. (62) reported that the polarization of anti-inflammatory, alternately activated M2 macrophages is CFTR dependent. In addition, MDMs from healthy controls treated with a CFTR inhibitor (CFTRInh-172) were incapable of polarizing into anti-inflammatory M2 macrophages, suggesting that understanding the link between CFTR dysfunction and M2 polarization may provide new therapeutic targets for CF (62). On the other hand, growing evidence supports that lncRNAs regulate CF. For example, McKiernan et al. (63) found that X inactivation-specific transcript (*XIST*) and *MALAT1* are differentially expressed in bronchial brushings of CF patients. Balloy et al. (64) also found that in *Pseudomonas aeruginosa* (PA)-infected CF bronchial epithelial cells, the expression of several lncRNAs, such as maternal expression gene 9 (*MEG9*) and bladder cancer-associated transcription 1 (*BLACAT1*), was downregulated. In addition, lncRNA *BGas* has been shown to be associated with CF by targeting and regulating CFTR directly (65). Unfortunately, current studies on the roles of lncRNA-mediated macrophage polarization in CF are limited and require further investigation. If macrophage function can be recovered by rectifying CFTR dysfunction *via* lncRNAs, lncRNAs will be promising in the control of the onset and progression of CF.

## 4 LncRNA-based regulation of macrophage polarization in interstitial lung disease

### 4.1 Idiopathic pulmonary fibrosis

IPF is a lethal, chronic, progressive interstitial lung disease characterized by the deposition of fibroblasts and collagen in the lung interstitum, resulting in destruction and fibrotic remodelling of lung tissue (66). M2 macrophages induce the differentiation of the myofibroblast phenotype by releasing the fibrogenic cytokine TGF-β1, contributing to the deposition of extracellular matrix, suggesting a key role in IPF (51, 67). A recent study showed that decreased M2 macrophage infiltration in the lung significantly protected mice from bleomycin-induced lung injury and fibrosis. These IPF pathogenic factors were further reported to be related to lncRNAs. For instance, Xiao et al. (26) found elevated *H19* expression levels and decreased let-7a expression levels in lung tissues from arsenite-induced pulmonary fibrosis mice. Mechanistically, *H19* acts as a ceRNA for let-7a regulating c-Myc, resulting in lower expression of M2 macrophage markers (CD206, Arg1, and TGF-β1) and lower fibrosis-related markers (p-SMAD2/3, SMAD4, α-SMA and collagen I) (26). Similarly, in the sera of arseniasis patients, *H19* levels were higher and let-7a levels were lower than those in healthy controls (26). These observations elucidate the possible mechanism of pulmonary fibrosis induced by arsenic exposure and provide a theoretical basis for its treatment. In addition to the TGF-β/Smad pathways, the macrophage-based pathways implicated in fibrosis also include Wnt/beta-catenin and PI3K-AKT-mTOR, which can be regulated by a number of lncRNAs, such as *GAS5* and *LOC102551149* (68–70), in other fibrosis-related diseases. Obviously, more investigations are required to understand whether these lncRNAs are pivotal regarding the regulation of macrophage polarization in IPF.

### 4.2 Sarcoidosis

Sarcoidosis is a granulomatous disease of unknown aetiology. Macrophages and CD4+ T cells play a key role in granuloma formation (71). A previous study showed that the proportion of M1 macrophages (defined as CD40 cell surface expression) in the pulmonary lumen of patients with sarcoidosis is significantly increased, while the proportion of M2 macrophages (defined as CD163 cell surface expression) tends to increase in nonspecific interstitial pneumonia (NSIP), IPF, and hypersensitivity pneumonia (HP) (72). In contrast, Shamaei and his colleagues reported enhanced CD163 staining in granulomas of patients with sarcoidosis compared with tuberculous granulomas (73). Using a one-sided M2 phenotype marker and different sample types may explain the contradiction between the above two studies. The mechanism of transition from acute inflammation (granuloma) to the fibrotic stage is particularly complex. Increasing evidence suggests that granuloma formation is supported by Th1/M1 immune polarization due to exaggerated TNF-α and INF-γ. In contrast to the acute phase of sarcoidosis, the chronic fibrotic disease state is associated with M2/Th2 polarization (74, 75). Thus, a key point in sarcoidosis therapeutics is restoring the M1/Th1 (inflammation) and M2/Th2 (fibrosis) balance. To date, the expression profile and functional studies of lncRNAs in sarcoidosis remain unknown. Considering the ability of lncRNAs to regulate macrophage polarization and participate in immune regulation, the diagnostic and therapeutic role of lncRNAs in sarcoidosis might be an important area of research.

### 4.3 Connective tissue disease-associated ILD

Approximately one-third of individuals with ILD have associated connective tissue disease (CTD) (76). The CTD demonstrating features of ILD include rheumatoid arthritis (RA), systemic lupus erythematosus (SLE), dermatomyositis (DM) and polymyositis (PM), systemic sclerosis (SSc), Sjogren’s syndrome (SS), and mixed connective tissue disease (MCTD) (77). RA-ILD is the most common type (78). RA is a chronic inflammatory autoimmune disease characterized by massive immune cell infiltration, pannus formation and destruction of cartilage and bone. Macrophages play a crucial role in the pathogenesis of RA, and the degree of synovial macrophage infiltration, particularly elevated M1 macrophages, correlates with clinical disease activity and severity in RA patients (79–81). Zhu et al. (82) found that lncRNA *H19* is upregulated in RA patients and arthritic mice. Additionally, *H19* overexpression promoted M1 macrophage polarization along with increased expression of M1 macrophage-related factors and aggravated arthritis in mice by upregulating KDM6A expression (82). Therefore, targeting *H19* may develop into a novel therapy for RA by inhibiting M1 macrophage polarization. As described previously, *H19* promotes myofibroblast differentiation in PF by regulating M2 polarization (26). It remains to be known whether *H19* is involved in the progression of pulmonary manifestations in RA by altering the macrophage phenotype.

In addition, the role of macrophage polarization and plasticity in SLE and DM/PM development has also been explored in several studies (83–85). Despite the emerging role of lncRNAs in autoimmune diseases, whether their regulatory role in these diseases is through the induction of macrophage polarization remains less understood and requires further exploration.

## 5 LncRNA-based regulation of macrophage polarization in infectious lung disease

### 5.1 Tuberculosis

Tuberculosis (TB) continues to be a major public health problem, with over 10 million new cases and 1.5 million deaths annually (86). *Mycobacterium tuberculosis* (MTB) is the most common pathogen causing TB and has high drug resistance (87). Macrophages play a crucial role in the host immune response and infection outcome post-TB infection (88). In addition, antibacterial activity and cytokine production during the formation of tuberculosis granuloma are usually concomitant with the transformation of the macrophage phenotype from M1 to M2 (88). Accordingly, MTB inhibit the development of the M1 phenotype and reprogram macrophages towards the M2 phenotype for better survival in the host, resulting in increased occurrence and development of pulmonary TB (89). It was proven that the fusion of lysosomes with TB-containing phagosomes and the upregulation of iNOS were caused by M1 polarization (90). Additionally, the TLR2/MyD88 signalling pathway associated with M1 macrophage activation plays a key role in host defence during MTB infection (91). Thus, inhibition of the intracellular survival of MTB may be orchestrated by M1 macrophage activation, which would facilitate pathogen clearance. Additionally, the protective effect of proinflammatory/M1 macrophages on MTB infection has been confirmed in many clinical studies (92). Although the administration of antibiotics has been widely used to prevent and treat TB, the persistence of latent infections and the emergence of resistance urgently require the development of new drugs and treatments. Therefore, controlling macrophage polarization is expected to improve TB progression.

Recent studies have identified ncRNA profiles and explored the functional role of lncRNAs such as *MEG3* and *NEAT1* during MTB infection, further implicating their potential in the immune response (93–95). Two lncRNAs are functionally related to polarized macrophages during MTB infection. For example, Luo et al. (27) discovered that *XIST* expression was upregulated in RAW264.7 cells and human monocyte-derived macrophages (hMDMs) after MTB infection and that its expression was regulated by ESAT-6, an important determinant of MTB virulence. Functionally, *XIST* serves as a competing endogenous RNA targeting miR-125b-5p, a miRNA that promotes M1 macrophage polarization by modulating A20/NF-κB signalling. A previous study also supported the conclusion that the expression of A20 was upregulated in MTB-infected macrophages, thereby inhibiting the NF-κB pathway and regulating the immune response after MTB infection (96). The regulatory network of the *XIST*/miR-125b-5p/A20/NF-κB axis has also been proven to be a molecular mechanism of negative pressure treatment for MTB infection (27). Another recent study reported that *MIR99AHG* is upregulated in M2 (IL-4/IL-13)-polarized mouse and human macrophages but downregulated after clinical MTB HN878 strain infection and in PBMCs from active TB patients (97). Knockdown of *MIR99AHG* using antisense oligonucleotides (ASOs) significantly reduced intracellular MTB growth, necrosis, and proinflammatory cytokine production in mouse and human macrophages, as well as reduced mycobacterial burden in the lungs of mice (28). Thus, as an addition to existing antibiotics, *MIR99AHG* may be a potential target for host-directed TB drug therapy.

### 5.2 Pneumonia

Pneumonia is the leading cause of death for children under the age of 5 (98). Particularly, after the outbreak of severe acute respiratory syndrome coronavirus type 2 (SARS-CoV-2) in Wuhan in December 2019, approximately 20-30% of patients hospitalized for COVID-19-associated pneumonia required intensive care for respiratory support (99, 100). At present, there are still difficulties in clinical treatment. Given that morbidity and mortality are associated with excessive inflammation, it is necessary to better understand the immunological basis of pneumonia in patients to better identify therapeutic targets. It has been shown that macrophages play an important role in pneumonia and polarize into different phenotypes when responding to various pathogens. For instance, macrophages are polarized towards an M1 phenotype in the early stage of bacterial infection and serve as a prompt to eliminate pathogens (101). As described by Li et al. (102), the M1 phenotype (iNOS^+^) was increased and the M2 phenotype (CD206^+^) was decreased after *Klebsiella pneumoniae*-induced pneumonia. High expression of IL-10 and a high population of M2-polarized macrophages play important roles in the production of lung consolidation in *Mycoplasma hyopneumoniae* infection (103). In SARS-CoV-2 infection, M1 macrophage-derived inflammatory cytokines, such as TNF-α and IL-1β, have been confirmed in the respiratory tract and are closely correlated with increased disease severity. M2 macrophages are thought to play an important role in the process of fibrosis (104–106). In addition, Shibata et al. reported that alveolar macrophages could acquire the M2 phenotype at Day 8 after RSV infection (107). During cryptococcal infection, the polarization status of pulmonary macrophages changed with time: at 1 week after infection, the pulmonary M2 macrophages were strongly polarized, but at 3 and 4 weeks after infection, the overall polarization of the macrophages shifted to M1. These studies suggest that the polarization of macrophages is phenotypic and functional plasticity shifts in response to changes in external stimuli. Targeting macrophage polarization and reinforcing phenotypic adaptation to the microenvironment may hold great promise for the treatment of pneumonia.

lncRNAs that regulate macrophage polarization may affect pneumonia. As an example, Chi et al. (29) found that *GAS5* mRNA expression was significantly decreased in hMDMs from children with pneumonia and this is accompanied by increased M2 phenotype macrophages compared with the control group, and *GAS5* overexpression promoted macrophage polarization from M2 to M1 in children with pneumonia *via* the miR-455-5p/SOCS3/JAK2/STAT3 axis, indicating its protective role in pneumonia in children. Indeed, the role of *GAS5* in the pathogenesis of pneumonia has been confirmed by emerging evidence (108–110). In addition, downregulation of lncRNA *GAS5* can decrease ACE2 expression by increasing miR-200c-3p and promote apoptosis of A549 cells, thus promoting the progression of acute respiratory distress syndrome (ARDS) (28, 111). It would be exciting if overexpressing lncRNA *GAS5* reduces the chance of death in severe viral pneumonia patients caused by ARDS.

### 5.3 ALI/ARDS

**Figure 4:**
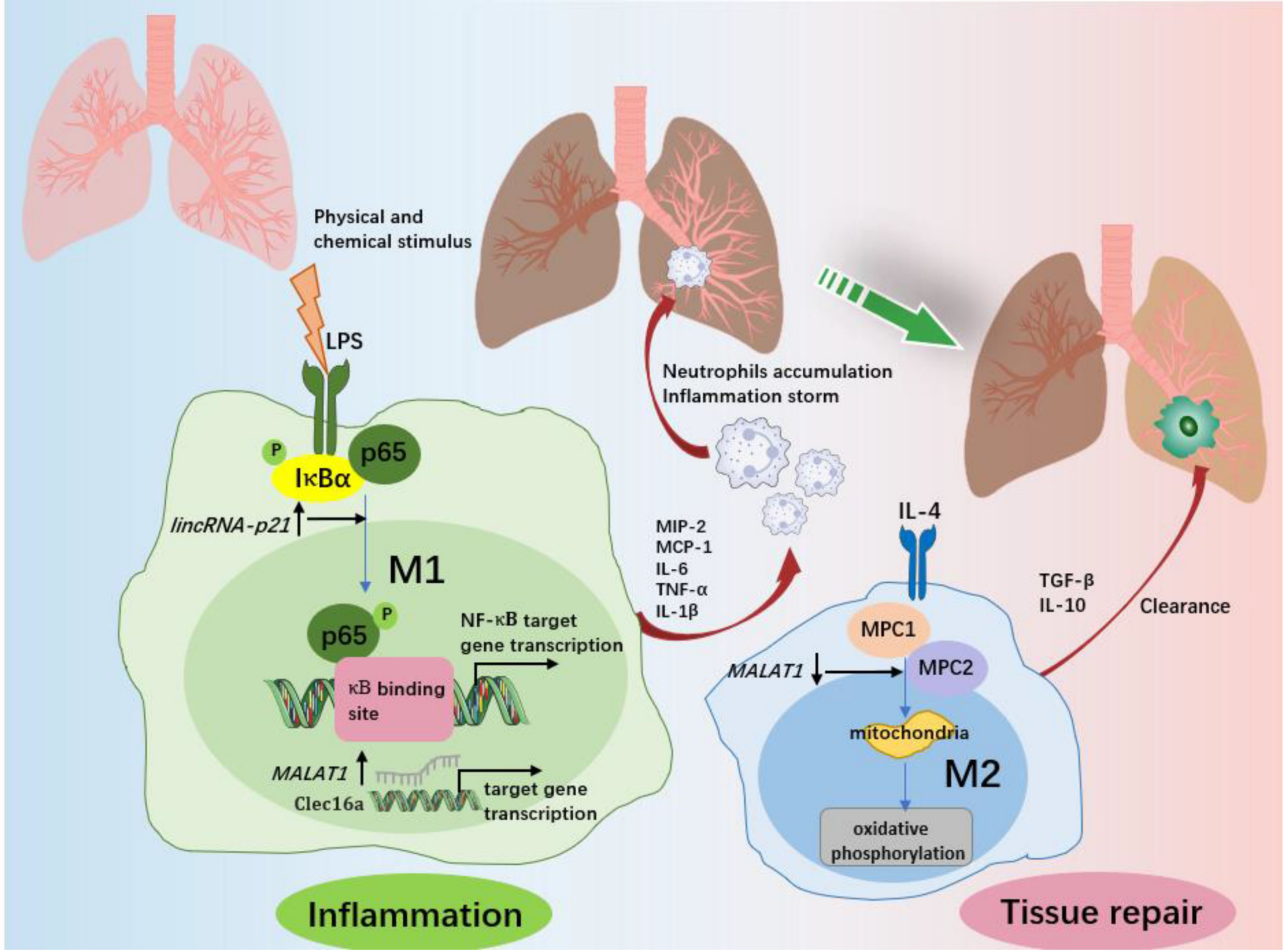
The role of macrophages and associated lncRNAs in ALI/ARDS. In response to various physicochemical stimuli, M1 macrophages release various proinflammatory cytokines at the site of inflammation and then recruit neutrophils from the circulation into the lung and alveolar spaces. Excess accumulation of proinflammatory factors and neutrophils promotes the progression of inflammation and lung injury. *LincRNA-p21* contributes to LPS-induced M1 activation by promoting the ubiquitination of IκBα, activating NF-κB as well as promoting p65 nuclear translocation. *MALAT1* promotes the expression of C-type lectin domain family 16, member A (Clec16a) in nucleus, which is required for the proinflammatory activation of M1 macrophages. After the elimination of the trigger, macrophages changed from the M1 to the M2 phenotype, and ALI/ARDS entered the recovery stage. M2 macrophages play an important role in inflammation resolution and lung tissue repair by limiting the levels of proinflammatory cytokines and enhancing the production of anti-inflammatory cytokines. *MALAT1* regulation of M2 activation of macrophages is dependent on glucose metabolism. Both mitochondrial pyruvate carriers (MPC)1 and MPC2 are upregulated by IL-4. IL-4 downregulates *MALAT1* expression, and *MALAT1* knockdown enhances the expression of MPC1 and MPC2, therefore regulating glucose-derived mitochondrial oxidative phosphorylation (OxPhos), which is essential to M2 macrophage polarization.

Acute lung injury (ALI) is a typical pathological feature of ARDS, and is characterized by the secretion of high levels of proinflammatory factors and the triggering of the inflammatory cascade, where alveolar macrophages (AMs), especially M1 macrophages, have been shown to be the major component (112, 113). Pulmonary fibrosis is the advanced stage of ALI/ARDS and is caused by fibroblast proliferation and excessive collagen deposition (12). In this phase, M2 phenotype macrophage-derived TGF-β and IL-10 play a leading role. Therefore, controlling macrophage polarization is expected to ameliorate the progression of ALI/ARDS. Several lncRNAs have been shown to be involved in inflammation-triggered ALI (**Figure 4**). For example, *lincRNA-p21* levels were highly expressed in AMs from LPS-induced ARDS mice in a time-independent manner, and *lincRNA-p21* inhibition reversed LPS-induced M1 activation and attenuated LPS-induced lung injury (21). Further experiments showed that *lincRNA-p21* overexpression promoted p65 nuclear translocation and NF-κB activity, indicating the underlying application of *lncRNA-p21* for ADRS therapy *via* NF-κB/p65-mediated pathways. Similar to *lincRNA*-*p21*, lncRNA *MALAT1* has the capacity to promote proinflammatory M1 activation and inhibit alternative M2 activation (30). Knocking out *MALAT1* reduced LPS-induced systemic and pulmonary inflammation and injury, but more severe bleomycin-induced pulmonary fibrosis and M2 alveolar macrophage augmentation were observed in mice (30). Mechanistically, *MALAT1* knockdown may promote mitochondrial pyruvate carriers (MPCs) and their mediated glucose-derived oxidative phosphorylation (OxPhos) induced by IL-4, thereby enhancing the M2 macrophage phenotype (114). This finding supports the role of *MALAT1* in promoting the progression of ALI. In addition, the silencing of *MALAT1* can reduce the inflammatory response in lung injury as well as the prevalence of cytokine storms in SARS-CoV-2 patients (115). It would be promising if targeting lncRNA *MALAT1* expression lowers the risk of COVID-19-related ARDS.

## 6 LncRNA-based regulation of macrophage polarization in lung cancer

Lung cancer, the most common cancer and the leading cause of cancer-related deaths, is mainly divided into two types: non-small cell lung cancer (NSCLC) and small cell lung cancer (SCLC) (116). NSCLC accounts for approximately 85% of lung cancer and is classified by histopathology as adenocarcinoma, squamous cell carcinoma and large cell carcinoma (117). Although molecular targeted therapies and immunotherapies are available for lung cancer patients, recurrence and progression due to drug resistance remain persistent problems (118). Currently, the modulation of immune responses in lung cancer has not been fully elucidated. Tumour-associated macrophages (TAMs), which are most likely of the M2 phenotype and have anti-inflammatory properties, play an important role in stimulating tumour cell proliferation and invasion as well as inducing tumour angiogenesis and lymphangiogenesis (119). Furthermore, M1 macrophages also play a key role in tumorigenesis. In the chronic inflammatory environment, M1 macrophages can not only induce the carcinogenic process by secreting proinflammatory mediators for a long time but also directly kill tumour cells and antagonize the growth of established tumours by stimulating the antitumour immune response (120). In this context, driving TAM polarization to modulate immune responses could be a novel therapeutic approach for lung cancer. A recent study discovered the functional relevance of lncRNAs in cancer immune regulation and TME, contributing to the progression and clinical outcome of a variety of cancers, including lung cancer (121, 122). Here, we discuss specific lncRNAs that regulate polarized TAMs in lung cancer.

Li et al. (31) reported that lncRNA *GNAS-AS1* was highly expressed in TAMs, human NSCLC cell lines and tumour tissues. Moreover, high *GNAS-AS1* expression predicts lower overall survival and metastasis-free survival of NSCLC patients (31). The oncogenic effect of *GNAS-AS1* is achieved by promotion of M2 polarization *via* directly inhibiting miR-4319, which can target the expression of N-terminal EF-hand calcium binding protein 3 (NECAB3) (31). Downregulation of NECAB3 in tumour cells has been shown to suppress tumorigenicity and play a crucial role in cancer development (123).

LncRNA *SNHG7* was highly expressed in docetaxel-resistant cells, and exosomal SNHG7 enhanced docetaxel resistance in lung adenocarcinoma (LUAD) cells by inducing autophagy and promoting M2 macrophage polarization (35). It was shown that lncRNA *SNHG7* activated the PI3K/AKT pathway by recruiting CUL4A to promote PTEN ubiquitination and thereby mediating macrophage M2 polarization (35). These discoveries implied that *SNHG7* may be a promising target for alleviating docetaxel resistance in LUAD.

Chen et al. (34) observed a higher expression of lncRNA *PCAT6* in human NSCLC cells, and siRNA-mediated knockdown of *PCAT6* inhibited the viability, migration, and invasion of NSCLC cells. A direct interaction was revealed by luciferase reporter assays between miR-326 and *PCAT6*. Kruppel-like Factor 1 (KLF1), an important participant in the process of macrophage polarization (124), is a direct target of miR-326. Consequently, *PCAT6* can activate KLF1 by sponging miR-326, induce macrophage M2 polarization and further promoting metastasis and EMT in NSCLC cells.

Wu et al. (33) demonstrated that CCL7 was abundantly expressed in LUAD and was associated with increased TAM infiltration. Additionally, CCL7 knockdown suppresses chemotactic migration and M2 macrophage polarization. Employing RNA immunoprecipitation and RNA pull-down assays, we found that *LINC01094* binds to SPI1 and promotes its nuclear translocation and that a luciferase reporter assay revealed an interaction between SPI1 and CCL7 (33). Therefore, *LINC01094* may be the cause of the aggregation of M2 macrophages and the spread of tumour cells caused by the upregulation of CCL7 in LUAD. However, the exact role of *LINC01094* in macrophage infiltration and LUAD development requires further investigation.

Another assessed lncRNA in lung cancer is lncRNA *LARRPM*. Li et al. (32) reported lower expression of *LARRPM* in LUAD tissues, which was negatively associated with poor survival and advanced stage in patients with LUAD. Further experiments showed that *LARRPM* could inhibit the proliferation, migration and invasion of LUAD cells, promote cell apoptosis, and inhibit M2 polarization and infiltration of macrophages by epigenetically regulating *LINC00240* and CSF1 (32). These results provide evidence of the potential utility of *LARRPM* as a prognostic biomarker and a therapeutic target for LUAD.

Collectively, these studies not only underscore the role of lncRNA-based TAM polarization in the pathogenesis of lung cancer but also identify lncRNAs as biomarkers for designing individualized treatment for patients with lung cancer.

## 7 Discussion

Macrophage polarization has functional significance in respiratory diseases, including nonneoplastic conditions and neoplastic conditions, by regulating inflammation, fibrosis, immune response, and tumorigenesis. Targeting macrophage phenotypic transformation may be a potential therapeutic strategy for respiratory diseases. With the rapid development of bioinformatics and high-throughput sequencing, diverse significant functional roles of lncRNAs in human diseases have been gradually revealed. A large number of studies have illustrated that lncRNAs are fundamental factors in genomic imprinting, chromatin modification, posttranscriptional regulation and transcription, splicing and modification and are involved in gene expression regulation at almost every stage in various diseases (125, 126). Recently, different lncRNAs have emerged as key regulators in the regulation of M1/M2 polarization. Given the important role of macrophage polarization in the development of respiratory diseases, the use of lncRNA-mediated M1/M2 polarization opens up new possibilities for the control of respiratory diseases.

Macrophage polarization plays a significant role in respiratory diseases, which are heterogeneous and dynamically evolving. For instance, M1 macrophages cause pulmonary inflammation in the early stages of ALI/ARDS, and M2 macrophages induce tissue repair and pulmonary fibrosis in the late stages. Th2-asthma is related to augmented M2 macrophages, while non-Th2 asthma is related to M1 macrophages. As described above, differentially expressed lncRNAs have the potential to serve as diagnostic or prognostic biomarkers of various respiratory diseases due to their abilities to regulate macrophage polarization. For example, several lncRNAs, including *AK085865*, *PTPRE-AS1*, and *lnc-BAZ2B*, have been demonstrated to be biomarkers for M2 macrophage-mediated Th2-asthma. *LARRPM* expression in lung tissues is negatively associated with advanced stage and poor survival in patients with lung cancer due to its ability to inhibit M2 polarization. Thus, lncRNAs that regulate macrophage polarization may be helpful indicators of respiratory disease stage and progression.

M1/M2 macrophage polarization is mediated by lncRNAs which directly target or sponge miRNAs to affect identified macrophage regulators and mediate respiratory disease development; therefore, targeting lncRNAs may become an effective therapeutic tool. For instance, the use of siRNA targeting *MIR155HG* reduced proinflammatory cytokines by shifting PBMCs of COPD patients from the M1 to the M2 phenotype. CRISPR/Cas9-mediated deletion of lncRNA *AK085865* ameliorates airway inflammation in asthmatic mice by inhibiting M2 macrophage polarization. The use of ASOs to knock out *MIR99AHG* inhibited the activation of M2 macrophages and significantly reduced the growth of MTB as well as the production of proinflammatory cytokines in the lungs of mice. In lung cancer, exosomal SNHG7 enhances docetaxel resistance in LUAD cells by inducing autophagy and promoting the polarization of M2 macrophages, which may provide clues for ways to reduce the likelihood of chemotherapy failure in lung cancer. In other words, using siRNA to downregulate *SNHG7* expression in exosomes that promote drug resistance is conducive to maintaining or recovering the sensitivity of cancer cells to chemotherapy drugs. To date, the role of lncRNA-mediated macrophage polarization in CF, sarcoidosis and CTD-ILD has not been explored. This could be a promising target for future exploration and verification.

Currently, RNA-based drugs have been approved for a variety of disease conditions, and many miRNA drug candidates are in clinical trials (i.e., TargomiR, an miR-16 mimic tested in mesothelioma, and Miravirsen, an miR-122 antagonist tested in HCV infection) (127–129). However, lncRNA-based therapy is still in its infancy, whether in animal studies or clinical trials. Direct delivery of lncRNA drugs to the lungs by inhalation is the most effective way to reduce systemic adverse effects. Gu et al. (126) demonstrated that intranasal delivery of shRNA lentivirus against *TUG1* blocks CS-induced inflammation and remodelling in a COPD mouse model. However, the stability of lncRNAs and delivery systems continues to present clinical challenges (130). In addition, the same lncRNA may mediate various biological processes by regulating multiple genes concurrently in response to different stimuli. The worry is that such indirect and complicated regulatory mechanisms make it difficult to target lncRNAs for therapeutic purposes. As an example, lncRNA *H19* promotes M1 macrophage polarization and aggravates arthritis by upregulating KDM6A expression (82), which contradicts the role of *H19* in promoting M2 polarization through the *H19*-miR let-7a/c-Myc axis in IPF. But for patients with rheumatoid arthritis complicated with IPF, targeting *H19* seems beneficial for disease progression. Therefore, compared with strategies targeting macrophage regulators or miRNAs, lncRNA-targeted therapy acting on polarization seems to be more difficult to implement and requires special precautions to minimize off-target adverse effects. Applying lncRNAs to drive effective reprogramming of macrophage polarization under specific disease conditions requires more effort. Most importantly, altering macrophage polarization might benefit a particular disease but exacerbate other coexisting diseases due to its heterogeneity and plasticity. For instance, *MALAT1*‐mediated M1 polarization is involved in different pulmonary processes and plays opposite roles in lung injury and pulmonary fibrosis. *PTPRE-AS1* deficiency exacerbates CRE allergen–induced lung inflammation and attenuates colitis in an acute DSS model by promoting M2 macrophages.

Despite promising in indicating the diagnosis, prognosis, or treatment of respiratory diseases, there are several obstacles to overcome regarding the clinical application. First, some lncRNAs such as *MIR155HG*, *lnc-BAZ2B*, and *LARRPM* studied in asthma, COPD, and lung cancer studies are based on a small sample size at present. Validation studies on larger sample numbers from multi-centers are required to identify those lncRNAs that could identify different disease stages and phenotypes of diseases, which might improve current disease diagnostic strategies and achieve individualized treatment. Furthermore, lncRNAs with therapeutic potential for certain respiratory diseases such as MTB, ALI/ARDS, and IPF are only being studied in animal models or cell lines. Further research is required to determine whether lncRNA can be effectively used in clinical settings. Second, as the disease progresses, the polarization of macrophages changes dynamically. For example, M1 macrophages cause pulmonary inflammation in the early stages of ALI/ARDS, and M2 macrophages induce tissue repair and pulmonary fibrosis in the late stages. Currently, we have only a very limited understanding of the regulation mechanisms between lncRNAs and highly heterogeneous macrophages. More studies should be done to clarify the role of lncRNAs in the progression of respiratory diseases *via* macrophage polarization. Third, the stability of lncRNAs in specimens needs more attention as it may be affected by the detection environment and specimen quality, storage time, and temperature. Besides the stability of lncRNAs, it is also imperative to address off-target adverse effects in order to optimize their efficacy. Finally, lncRNAs’ economic benefits and broad applicability should also be considered.

## Conclusion

The present study suggests that manipulation of lncRNA expression can be used as a novel modality to regulate macrophage polarization, thereby regulating inflammation, fibrosis, immune response, and tumorigenesis in the respiratory system. Accumulating evidence has demonstrated that lncRNAs have the potential to become diagnostic or prognostic biomarkers and therapeutic targets in COPD, asthma, lung cancer, IPF, pneumonia, and ALI/ARDS. However, explorations of the roles of lncRNAs related to macrophage polarization in CF, sarcoidosis, and CTD-ILD are still lacking. Further confirmatory studies are essential to broaden the lncRNA horizon for the proper elucidation of novel lncRNAs that are likely to emerge as important regulators of macrophage polarization in broad respiratory diseases. Hopefully, lncRNA discovery will complement macrophage-centered diagnostic and therapeutic strategies allowing them to be used in the clinic more quickly.

## Author contributions

YY, WW, QW, and JK designed, supervised, and critically revised the manuscript. XQ drafted the manuscript. YD, DW and AZ did the reference collection. All authors contributed to the article and approved the submitted version.
